# Complexity of Running and Its Relationship with Joint Kinematics during a Prolonged Run

**DOI:** 10.3390/ijerph19159656

**Published:** 2022-08-05

**Authors:** Siddhartha Bikram Panday, Prabhat Pathak, Jeheon Moon, Dohoon Koo

**Affiliations:** 1 Department of Sport and Leisure Studies, Keimyung University, Daegu 42601, Korea; 2 Department of Physical Education, Seoul National University, Seoul 08826, Korea; 3 Department of Physical Education, Korea National University of Education, Cheongju-si 28173, Korea; 4 Department of Exercise Prescription, College of Medical Science, Jeonju University, Jeonju 55069, Korea

**Keywords:** complexity, detrended fluctuation analysis, variability, elite runners

## Abstract

We investigated the effect of prolonged running on joint kinematics and its association with stride complexity between novice and elite runners. Ten elite marathoners and eleven healthy individuals took part in a 20 min submaximal prolonged running experiment at their preferred running speed (PRS). A three-dimensional motion capture system was utilized to capture and calculate the alpha exponent, stride-to-stride fluctuations (SSFs), and stride-to-stride variability (SSV) of spatiotemporal parameters and joint kinematics. In the results, the elite athletes ran at a considerably higher PRS than the novice runners, yet no significant differences were found in respiratory exchange ratio with increasing time intervals. For the spatiotemporal parameters, we observed a significant increase in the step width and length variability in novice runners with increasing time-interval (*p* < 0.05). However, we did not observe any differences in the alpha exponent of spatiotemporal parameters. Significant differences in SSF of joint kinematics were observed, particularly in the sagittal plane for ankle, knee, and hip at heel strike (*p* < 0.05). While in mid-stance, time-interval differences were observed in novices who ran with a lower knee flexion angle (*p* < 0.05). During toe-off, significantly higher SSV was observed, particularly in the hip and ankle for novices (*p* < 0.05). The correlation analysis of joint SSV revealed a distinct negative relationship with the alpha exponent of step-length and step-width for elite runners, while, for novices, a positive relation was observed only for the alpha exponent of step-width. In conclusion, our study shows that increased step-width variability seen in novices could be a compensatory mechanism to maintain performance and mitigate the loss of stability. On the other hand, elite runners showed a training-induced effective modulation of lower-limb kinematics to improve their running performance.

## 1. Introduction

The complexity of the human gait comprehends the locomotor adaptability and flexibility of the system. Even in a steady state, the gait pattern fluctuates as it requires optimal coordination of several degrees of freedom while conforming to internal and external perturbations [1,2,3]. Traditionally, linear analysis methods have been used to assess the gait, mainly analyzing either the central tendency or variability of spatiotemporal parameters and joint kinematics [4]. However, human locomotion is stochastic and inherently non-linear, making non-linear methods more intuitive for understanding the fundamental mechanism of the locomotor system [5,6]. One of the widely utilized non-linear methods to assess the complexity of time-evolving gait behavior is detrended fluctuation analysis (DFA) [2,6,7]. The method scales the long-term autocorrelations of non-stationary gait signals and quantifies changes in the time series by comparing its self-similarity to a value of the fractal scaling index “alpha”.

Unlike walking, the gait cycle in running consists of a sequence of parabolic aerial phases that require first braking upon ground contact and then generating propulsion impulses one step at a time from the ground [8]. Thus, efficient running is achieved with proper utilization of the spring-mass mechanism of the lower limb joints that act as pseudo springs to propel the body forward [9]. Particularly, trained distance runners have been reported to improve their running economy by using pacing strategies, which involve modulating their whole-body joints and segments to maintain a self-selected speed, i.e., preferred speed and intensity [10,11,12]. In previous research, pacing strategy has been assessed using performance parameters such as marathon time or speed, which are dependent on stride length or frequency [10]. According to studies, elite marathon runners have much less variability in their speed and stride intervals during a marathon run than novice runners [2,13,14]. This has led to studies postulating that the strategy of minimizing performance variability of spatiotemporal parameters by elite runners is achieved by reducing joint variability throughout the run because of the training regimen that fosters stable and consistent motor patterns [2,14,15].

The end-point variability of the spatiotemporal parameters (e.g., stride length, stride time) has been reported to decrease as runners increase their performance through training [2]. Although quantifying performance using spatiotemporal features (i.e., stride characteristics) provides valuable information on running mechanics, it does not provide a comprehensive understanding of the underlying mechanisms behind movement generation. High kinematic variability of joints/segments, on the other hand, allows for flexibility in the movement pattern and may help in goal-directed performance against any perturbation [16,17,18]. Hence, the evaluation of the central tendency, i.e., stride-to-stride fluctuation (SSF) and variability, i.e., stride-to-stride variation (SSV) of the lower extremities, is essential to understand the running dynamics [19,20,21]. Furthermore, the majority of running-related studies have focused only on the final motor-put, i.e., spatiotemporal parameters, and examined the effects of speed, skill level, or running surface only during short running trials [2,18,20,21,22,23,24]. Those that utilized non-linear methods such as DFA to analyze complexity did not shed light on the role of joint kinematics in interpreting the complexity of running [2,21,25].

Therefore, we still lack a complete understanding of (i) the influence of prolonged running on central tendency and variability of spatiotemporal and joint kinematics in running, (ii) the differences in kinematics between elite and novice runners, and (ii) their association with complexity. To this end, we evaluated the alpha exponent, SSFs, and SSV of spatiotemporal and joint kinematics between novice and elite runners during running over increasing time-periods for long-term running trials.

## 2. Materials and Methods

### 2.1. Participants

Twenty-one healthy men were recruited for the study; ten marathoners categorized as the elite athlete group (age: 28 ± 4 yrs., height: 1.76 ± 0.07 m, weight: 66 ± 10 kg, and a half marathon record of 1 h 14 min ± 6.35 min, experience 8.60 ± 4.40 yrs.) and eleven young and healthy participants categorized as the novice group (24 ± 3 yrs., height: 1.80 ± 0.06 m, and weight: 76 ± 8 kg) (details in Appendix A). For each group, the sample size required to achieve statistical significance was determined to be eight. To calculate the sample size, we referred to a study that compared elite and novice runners’ running efficiency (RE) and used the mean difference in running energy cost to conclude the effect size to be 1.97 [22]. Thereafter, the G*Power program (3.1.9.7, Heinrich Heine University, Düsseldorf, Germany) was used to input the aforementioned settings, with the statistical significance set to 0.05 and the statistical power set to 0.95 [26].

The inclusion criteria for the elite athlete group were a regular marathon runner with at least five years of experience, having a weekly distance of more than 40 km, and being able to run on a treadmill. The novice category includes anyone who regularly engages in aerobic exercise for at least 2–4 h per week. The existence of any neurological, ocular, vestibular, orthopedic, or muscular disorders that might affect running ability was an exclusion criterion. All aspects of the study were carried out in accordance with the Helsinki Declaration’s principles and ethical standards, and with the consent of Seoul National University’s International Review Board (IRB: 2010/003-013). The individuals were informed of the experimental method as well as the risks involved. Furthermore, before taking part in the study, the individuals provided their written consent.

### 2.2. Experimental Equipment

The participants took part in a running experiment on top of a customized treadmill (specification: length (2.5 m), breadth (1.2 m), height (0.25 m), horsepower (5 hp.), maximum speed (30 km/h), maximum inclination (20 percent) (Any Fitness, Ltd., Gwangju, Korea). Twelve infrared cameras (Optitrack Prime13, NaturalPoint, Inc., Corvallis, OR, USA) were used to capture the lower extremity joint kinematics at a sampling frequency of 100 Hz. The *X*-, *Y*-, and *Z*-axes of the motion capture system represented the coordinates of the retro-reflective markers in the medial-lateral, anterior-posterior, and vertical directions, respectively [27].

An indirect calorimetry device (K5, Cosmed, Rome, Italy) was used to assess the metabolic expenditure using the breath-by-breath analysis approach to measure oxygen inhalation and carbon dioxide exhalation. The device was calibrated using a known concentration of oxygen and carbon dioxide through a reference gas cylinder and its standard error of measurement has been reported to be 1.6 percent for the rate of oxygen intake and 2.2 percent for the rate of carbon dioxide exhalation [28].

### 2.3. Experimental Protocol

The experimental procedure utilized was similar to the doctoral thesis of the first author [29]. Participants were advised over the phone to avoid engaging in any activities that may increase exhaustion for at least 24 h prior to the experiment. When the participants arrived at the laboratory, they were given a detailed overview of the aim, experimental methodology, and possible risks, and were asked to provide written informed consent. A brief interview was conducted for participants to discuss their eligibility, criteria, demographic data, physical activity participation level, running history, and completion of the modified Waterloo Footedness Questionnaire. Then, anthropometric data such as age, height, weight, and shoe size were also gathered, and an athletic attire was provided for the experiment. To reduce the risk of injury during the experiment, a warm-up session was conducted which included participants walking on the treadmill for 10 min at a self-selected pace, followed by a 5-min rest period, and a 10-min whole-body stretching exercise. In the meantime, the age-predicted maximum heart rate (maxHR) (220-age) of each participant, heart rate reserve (HRR), and submaximal heart rate range (70~85%) were calculated for reference [30].

First, our study required participants to run at their comfortable or preferred running speed (PRS). We referred to the PRS estimation method utilized by previous studies [25,31] and modified based on our requirement. A detailed explanation has been described in Appendix B.1. After estimating the PRS and conducting a familiarization run, participants rested for at least 10 min. Thereafter, the metabolic system and HR were refitted to the participant to monitor in real-time whether they stayed within their submaximal level of the anaerobic threshold percentage (respiratory exchange ratio; RER less than 1.0) and maintained their performance throughout the run (Appendix B.2) [32,33]. For kinematic analysis, 34 spherical retro-reflective markers (diameter: 12.7 mm) were attached to the anatomical landmarks of the joints and segments of the lower extremity, and a static standing calibration trial in the anatomical position of 3 s was recorded. (Details in Appendix B.3). Then, a typical 8 min procedure for O_2_ consumption at a steady state was measured, consisting of 5 min of sitting and 3 min of standing.

The running experiment started with the subjects walking for at least 3 min at their own pace. The experimenter then gradually increased the treadmill speed over 5 min to their respective PRS. The treadmill dashboard was covered so that subjects avoided making any unnecessary movements. After completing the 20 min run, the experimenter progressively reduced the treadmill speed to the prior self-selected walking speed of the subject and 5 min of walking was performed as cool down. Throughout the trial, HR and RER were constantly monitored in real-time to ensure that they did not overexert themselves and that their HR stayed stable and stayed below their maxHR. The experiment was terminated if the subjects reached their maxHR or upon request.

### 2.4. Data Analysis

Using Visual 3-D software (v6, C-Motion Inc.: Germantown, MA, USA), the coordinates of the retro-reflective markers were processed. First, a zero-lag second-order low-pass Butterworth filter with a cut-off frequency of 10 Hz was used to filter the raw coordinates of the retro-reflective markers [34,35]. The coordinates of the retro-reflective markers from the static standing trial were used to construct a whole-body kinematic model. The pelvis, bilateral thighs, shanks, and feet were among the seven skeletal segments included in the kinematic model, and the joint center locations were estimated using a mid-point estimation approach [36]. The local coordinate system of each segment was based on the Cardan sequence (x-y-z).

#### 2.4.1. Calculation of Spatiotemporal and Kinematic Variables

A running gait cycle consists of three distinct phases: (i) a stance, (ii) a swing phase, and (iii) a flight phase. The initial half of the stance phase is dedicated to force absorption, while the second half is dedicated to propulsion [37]. Each stance phase can be subdivided into events, i.e., initial contact or heel strike (HS) corresponding to braking, midstance (MS) corresponding to balance, and toe-off (TO) corresponding to propulsion. In our study, we utilized an algorithm developed by Zeni Jr, et al. [38] to detect the events of the stance phase (HS and TO) using the Y-direction relative trajectory of the coordinates of the heel marker and the COM of the whole body. When it is plotted against the time, the Y-direction relative trajectory forms a sinusoidal curve, and the time points of the HS are the peak points of the sinusoidal curve, while the valleys are toe-off (TO). The MS was defined as the point at which the y-axis coordinate of the ankle COM of the supporting limb aligns with the y-axis coordinate of the ankle COM of the swinging limb. Thus, based on the events of the stance phase (HS, MS, and TO), we calculated spatiotemporal and joint kinematics across 20 min of prolonged run. The average and standard deviation of the spatiotemporal and joint kinematics are taken as stride-to-stride fluctuation (SSF) and stride-to-stride variation (SSV), respectively.

- Spatiotemporal variables: include step time in seconds (s), step length, and step width in centimeters (cm).
- Joint Kinematics: Similarly, the joint angles of the ankle, knee, and hip were computed based on the Cardan sequence, where the *X*-, *Y*-, and *Z*-axes corresponded to flexion/extension, abduction/adduction, and internal/external rotation angles, respectively. Thereafter, discrete joint angles were assessed, including hip, knee, and ankle angles, in sagittal (*X*), frontal (*Y*), and transverse (*Z*) planes for each event of the stance phase. The units are expressed in degrees (°).

#### 2.4.2. Calculation of Running Complexity

To calculate the running complexity, DFA was utilized, which yields a scaling exponent that describes the fractal-like structure present in the locomotor control system [2,6,21,25]. The DFA algorithm was implemented based on the algorithm described by Peng, et al. [39]. Initially, we calculated an accumulated sum of the time series and divided it into equal windows, where a total number of stride intervals (*N*) were converted into random walks by subtracting the mean value and integrating to generate a time series ST as:

(1) $$ST_{int}\left(k\right)=\overset{q}{\underset{i=1}{\sum }}\left[ST\left(i\right)-ST_{avg}\right]\;\;\left(q=1,2,\ldots .,N\right),$$

where ST(i) is the $i^{th}$ stride interval and $ST_{avg}$ is the average stride interval. Then, the new integrated time series $ST_{int}\left(k\right)$ was divided into non-overlapping boxes of equal length of *n* samples and a local least-squares line was fitted to the data, i.e., $ST_{det_{n}}\left(k\right)$ for each box. Subsequently, the average fluctuation of data $ST_{int}\left(k\right)$ with respect to the line, which is the residual variance, was calculated for each box size n as:

(2) $$F\left(n\right)=\sqrt{\frac{1}{N}\overset{N}{\underset{k=1}{\sum }}{\left[ST_{int}\left(k\right)-ST_{det_{n}}\left(k\right)\right]}^{2}}.$$

Following the suggestion in previous studies [21,39], the window size was set to 4:4:*N*/4. Typically, F(n) will increase with increasing n, and the slope of the linear square fitted straight line between the F(n) and *n* on a log–log plot indicates the presence of power-law scaling, which is the scaling exponent alpha (α) [21,39,40,41]. An alpha value of 0.5 indicates the uncorrelated white noise, 1.5 for Brownian noise, and a value between 0.5 and 1 for time series indicates a long-range correlation [42]. The value of alpha > 0.5 indicates a tendency of statistical persistence, i.e., the longer stride interval in the past is likely to be followed another longer interval. Whereas, alpha < 0.5 indicates anti-persistence, i.e., longer stride in the past is likely to be followed with shorter stride intervals [6,43]. The scaling exponent alpha was calculated for spatiotemporal parameters such as, step-time (*α^Time^*), step-length (*α^Length^*) and step-width (*α^Width^*) for each 5 min interval during the prolonged run. All the analysis was conducted using a custom script written in Matlab 2021 (MathWorks, Inc., Natick, MA, USA).

### 2.5. Statistics

The mean and standard deviation of each variable was calculated for every five minutes intervals (P1: 0 to 5 min; P2: 5 to 10 min; P3: 10 to 15 min; and P4: 15 to 20 min). To investigate the main effects and interactions of these parameters for each of the spatiotemporal and kinematic variables of SSFs, SSV, and DFA, we utilized a 2 × 4 (two groups, four time intervals) mixed model analysis of variance (ANOVA). Furthermore, one-way ANOVA was used to evaluate the difference between the two groups, while the paired *t*-test was performed to identify any significant difference between time periods for each group. Mauchly’s sphericity test was used to examine the assumption of sphericity; if violated, Greenhouse–Geisser was employed to minimize the degrees of freedom. Thereafter, Pearson correlation was conducted to examine the relationship between the alpha exponent of the DFA of spatiotemporal variable and SSV of joint kinematics. The scale utilized to interpret the correlation coefficient was 0~0.19: very low correlation, 0.2~0.39: low correlation, 0.4~0.59: moderate correlation, 0.6~0.79: high correlation and 0.8~1.0: very high correlation. All tests were carried out using statistical software (SPSS version 25.0, IBM Inc., Chicago, IL, USA). The threshold for statistical significance was set at *p* < 0.05.

## 3. Results

Participants from each group performed the running task at their PRS. The independent sample *t*-test for PRS reported significant differences between groups, with elite athletes (M = 3.786 m/s, SD = 0.178 m/s) performing at significantly higher speed than novice runners (M = 2.828 m/s, SD = 0.265 m/s) [*t*_(19)_ = 9.595, *p* < 0.001]. For RER between elite and novice runners, the mixed-model ANOVA revealed no significant main effect of group or time-interval, and no significant group × time-interval interaction.

### 3.1. Effect of Prolonged Running on SSFs and SSV of Spatiotemporal Variables

**Figure 1:**
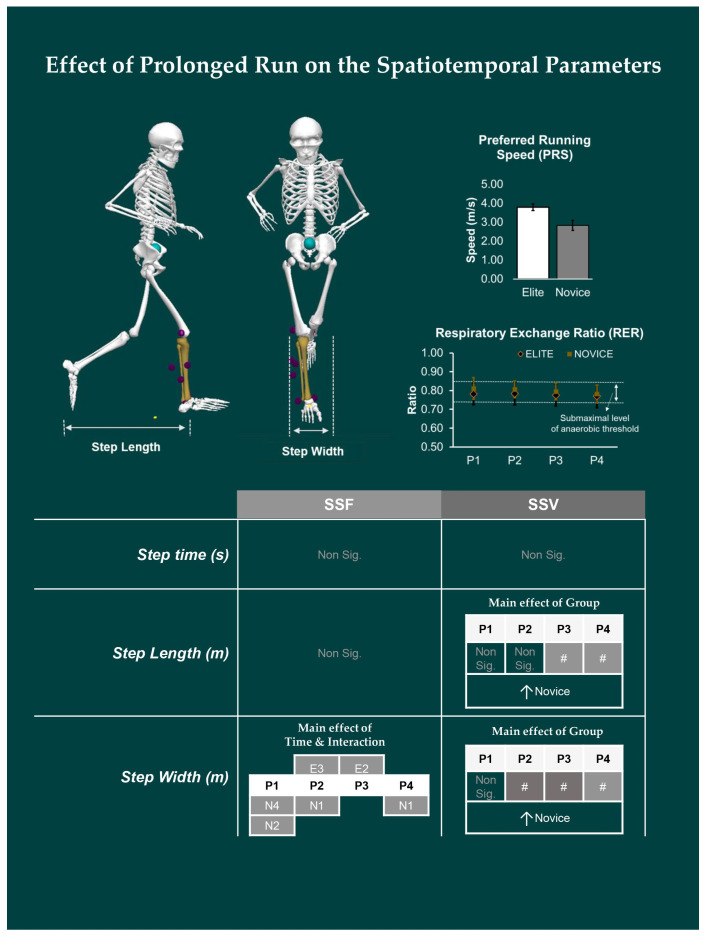
The change in spatiotemporal variables between novice and elite runners throughout the prolonged run is presented here. Note: The main effect of Group and Interaction represented by “#”. The main effect of Time and Interaction for each time interval for each group (E = Elite; N = Novice), for example, the difference in time interval in P1 vs. P2 for novices is denoted as N1 vs. N2. Non-significant differences are represented by “Non sig.”. For details, see Appendix C. The skeleton used for figure was created using Visual3D software.

The SSFs and SSV of the spatiotemporal variables for elite and novices during the prolonged run is shown in Figure 1. For SSFs, the mixed-model ANOVA revealed only a significant main effect of time-interval [*F*_(1.995, 37.913)_ = 5.917, *p* = 0.005]. The pair-wise comparison revealed significant differences in the *step-width^SSF^* for P2 vs. P3 time-interval for elite athletes. In contrast, for the novices, significant differences were observed for P1 vs. P2 and P1 vs. P4 time-interval.

For the SSV, the only significant effect of this group was observed for the *step-length^SSV^* [*F*_(1, 19)_ = 5.449; *p* = 0.031] and *step-width^SSV^* [*F*_(1, 19)_ = 8.132; *p* = 0.010]. The independent-samples *t*-test showed that elite athletes had significantly lower *step-length^SSV^* and *step-width^SSV^* at the P3 and P4 time intervals, and P2, P3, and P4 time intervals than novice, respectively.

### 3.2. Effect of Prolonged Running on SSFs of Kinematic Variables

**Figure 2:**
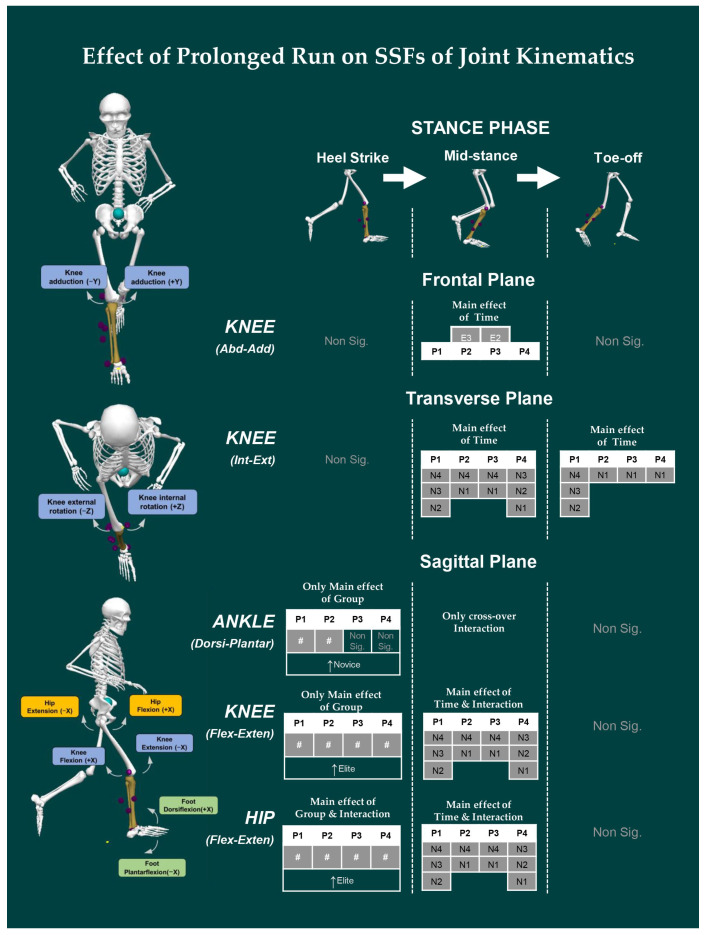
The change in SSFs for joint angles of lower extremity between novice and elite runners throughout the prolonged run is presented here. Note: Only results with significant differences are labeled in the skeleton. “Stride-to-stride fluctuation” denoted as: “SSF” and “stride-to-stride variation” denoted as “SSV”, The main effect of Group and Interaction represented by “#”. The main effect of Time and Interaction for each time interval for each group (E = Elite; N = Novice), for example, the difference in time interval in P1 vs. P2 for novices is denoted as N1 vs. N2. Non-significant differences are represented by “Non sig.” For details, see Appendix C. The skeleton used for figure was created using Visual 3D software.

The SSFs of the three-dimensional kinematic variables of the lower extremities for elites and novices during the prolonged run is shown in Figure 2. For *ankle^SSF^*, a significant main effect of the group was observed for *ankle^Dorsi-Plantar^* at the HS [*F* _(1, 19)_ = 4.578, *p* = 0.046] in P1 and P2 time-interval. At MS, there was a significant crossover interaction effect only for *ankle^Dorsi-Plantar^* [*F*_(1.315, 24.987)_ = 4.499, *p* = 0.035]. At TO, no significant main effect was observed for the group, time-interval, or group × time-interval interaction.

For *knee^SSF^*, a significant main effect of group was observed at HS for *knee^Flex-Exten^* [*F* _(1, 19)_ = 14.176, *p* = 0.001] at P1, P2, P3, and P4 time-intervals. At MS, there was a significant interaction effect for *knee^Flex-Exten^* [*F* _(1.529, 29.055)_ = 4.206, *p* = 0.034], with the main effect of time-interval [*F* _(1.529, 29.055)_ = 5.897, *p* = 0.012; novice: P1 vs. P2, P3 and P4; P2 vs. P4, P3 vs. P4]. In addition, at MS, the main effect of time-interval was also significant in *knee^Abd-Add^* [*F* _(1.101, 20.926)_ = 4.528, *p* = 0.042; elite: P2 vs. P3] and for *knee^Int-Ext^* [*F* _(1.190, 22.606)_ = 6.080, *p* = 0.017; novice: P1 vs. P2, P3, and P4; P2 vs. P3 and P4; P3 vs. P4]. At TO, a significant main effect of only the time-interval was observed for *knee^Int-Ext^* [*F* _(1.195, 22.701)_ = 5.524, *p* = 0.023; novice: P1 vs. P2, P3 and P4].

For *hip^SSF^*, a significant main effect of only group was observed at HS for *hip^Flex-Exten^* [*F* _(1, 19)_ = 12.669, *p* = 0.002] at P1, P2, P3, and P4 time-interval with a significant interaction effect [*F* _(1.986, 37.733)_ = 8.447, *p* = 0.001]. At MS, there was a significant main effect of only time-interval for *hip^Flex-Exten^* [*F*_(1.473, 27.989)_ = 6.355, *p* = 0.010] with a significant interaction effect [*F* _(1.473, 27.989)_ = 5.749, *p* = 0.014]. At TO, no significant effect was observed on group, time, or group × time-interval.

### 3.3. Effect of Prolonged Running on SSV of Kinematic Variables

**Figure 3:**
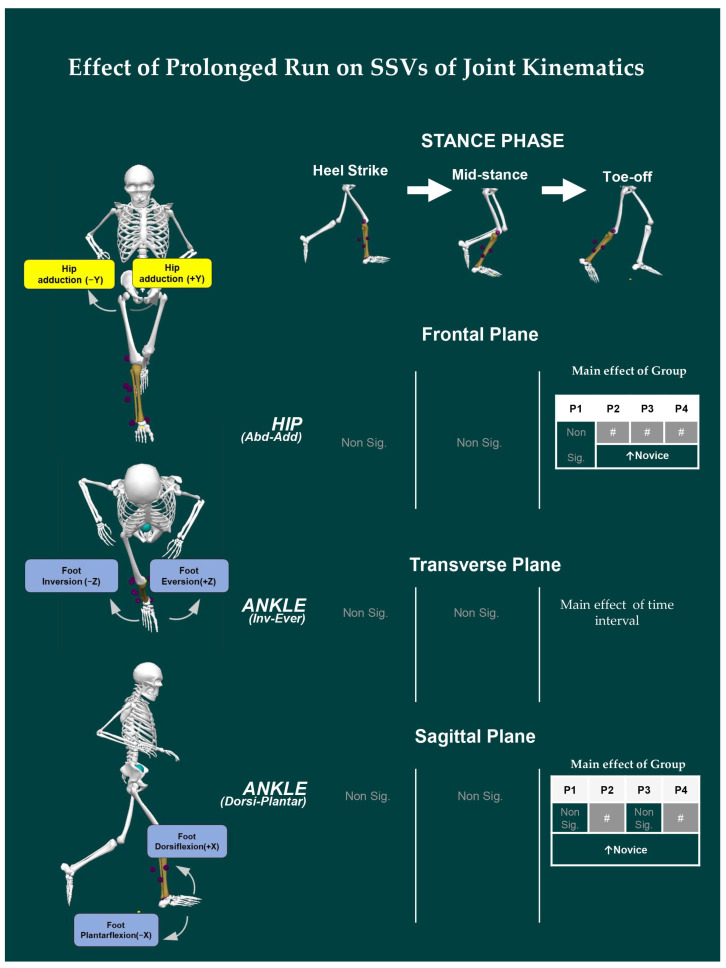
The change in SSV for joint angles of lower extremity between novice and elite runners throughout the prolonged run is presented here. Note: Only results with significant differences are labeled in the skeleton. “Stride-to-stride fluctuation” denoted as: “SSF” and “stride-to-stride variation” denoted as “SSV”. The main effect of Group and Interaction represented by “#”. The main effect of Time and Interaction for each time interval for each group, for example, the difference in time interval in P1 vs. P2 for novices is denoted as N1 vs. N2. Non-significant differences are represented by “Non sig.” For details, see Appendix C. The skeleton used for figure was created using Visual 3D software.

The SSV of the three-dimensional kinematic variables of the lower extremities for elites and novices during the prolonged run is shown in Figure 3. For *ankle^SSV^* at the TO, a significant main effect of the group was observed in *ankle^Dorsi-Plantar^* [*F* _(1, 19)_ = 4.583; *p* = 0.045] in P2 and P4 time-interval. Similarly, a significant main effect of only time-interval was also observed for *ankle^Inv-Ever^* [*F*_(2.012, 38.221)_ = 3.317; *p* = 0.047].

For *knee^SSV^*, no significant effects on the group, time, as well as group × time-interval were observed in all three events, i.e., HS, MS, and TO.

For *hip^SSV^,* a significant main effect of group was observed at TO for *hip^Abd-Add^* [*F* _(1, 19)_ = 5.685, *p* = 0.028] at P2, P3, and P4 time-intervals, with a non-significant time-interval and group × time-interval interaction effect.

### 3.4. Effect of Prolonged Running on Complexity

**Figure 4:**
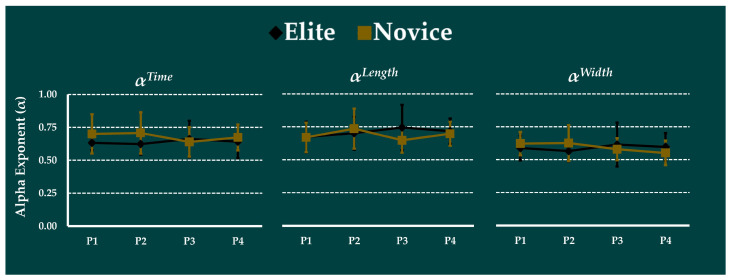
The effect of prolonged run in the alpha exponent of spatiotemporal parameters between novice and elite runners throughout the prolonged run is presented here. Alpha exponent for step-time as *α^Time^,* alpha exponent for step-length *α^Length^*, and alpha exponent for step-width as *α^Width^*. Elite denoted as black diamond, and Novice denoted as khaki square. No significant differences were observed between groups or time-intervals.

The alpha exponent of the spatiotemporal variables for elite and novice runners during the prolonged run is shown in Figure 4. The mixed-model ANOVA revealed no significant main effects of group or time-interval, and no significant group × time-interval interaction.

### 3.5. Correlation Analysis of Complexity with Joint Kinematics SSVs

**Figure 5:**
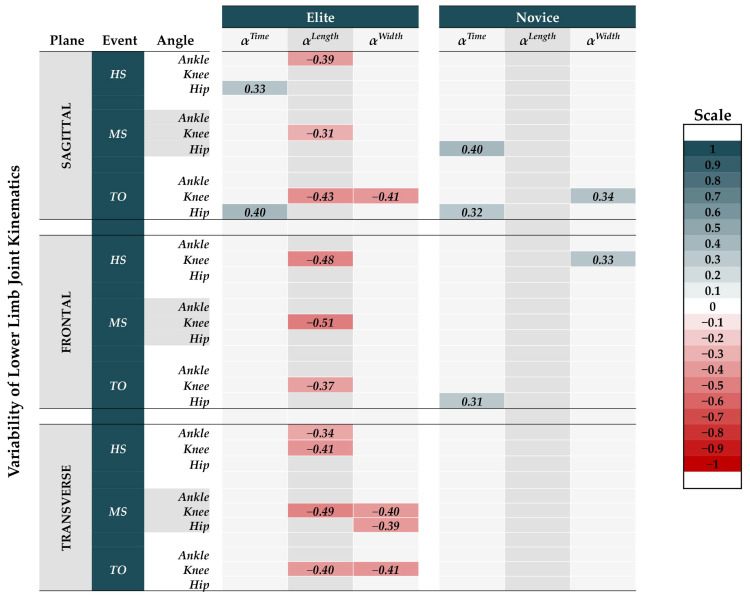
The results of the Pearson correlation analysis for the alpha exponent of spatiotemporal parameters with the variability of joint kinematics for elite and novice runners are presented above.

The Pearson correlation was computed to assess the relationship between complexity and joint kinematics SSVs for elite and novice runners separately (Figure 5). In elite runners, the *α^Time^* had significant positive correlation only with *hip^Flex-Exten^* angle at HS and TO. On the other hand, the *α^Length^* was negatively correlated with *ankle^SSV^* and *knee^SSV^*. For the *ankle^SSV^* at HS, the *ankle^Dorsi-Plantar^* angle and the *ankle^Inv-Ever^* angle had significant negative correlations with *α^Length^*. For the *knee^SSV^* at HS, the *knee^Add-Abd^* angle and the *knee^Int-Ext^* angle both had significant negative correlation with *α^Length^*. For the *knee^SSV^* at MS, significant negative correlation was observed in all three planes with *α^Length^*. The *knee^Flex-Exten^*, *knee^Add-Abd^*, and *knee^Int-Ext^* angles had significant negative correlation with *α^Length^*. For the *knee^SSV^* at TO, significant negative correlation was observed in all three planes with *α^Length^*. The *knee^Add-Abd^*, *knee^Flex-Exten^*, and *knee^Int-Ext^* angles had significant negative correlation with *α^Length^*.

For the *α^Width^* in elite runners, *knee^SSV^* had significant negative correlation with the *knee^Int-Ext^* angle at MS. Furthermore, at TO, significant negative correlation was observed for *knee^Flex-Exten^* and *knee^Int-Ext^* angles with *α^Width^*. For *hip^SSV^* at MS, *hip^Int-Ext^* angle had significant negative correlation with *α^Width^*.

In novice runners, the *α^Time^* had correlation only with *hip^SSV^*. At MS, the *hip^Flex-Exten^* angle had significant positive correlation with *α^Time^*. At TO, *hip^Flex-Exten^* and the *hip^Abd-Add^* angles both had significant positive correlation with *α^Time^*. For the *α^Width^*, significant positive correlation was only observed with the *knee^SSV^*. The *knee^Flex-Exten^* angle at TO and the *knee^Add-Abd^* angle at HS both had significant positive correlation with *α^Width^*.

## 4. Discussion

The purpose of our study was to assess the effect of prolonged running on joint kinematics and its relationship with the indices of complexity between novice and elite runners. We observed significant difference only in *step-width^SSF^* across time intervals for both elite and novice runners. Group differences in variability were observed for both *step-length^SSV^* and *step-width^SSV^*, such that novices exhibited greater step-to-step variability than elites. However, the complexity indices computed using DFA yield no significant main effect of group or time-interval or interaction. Similarly, joint SSFs (*ankle^Dorsi-Plantar^*, *knee^Flex-Exten^*, and *hip^Flex-Exten^*) were significantly different between groups at HS. Whereas time-interval differences were observed in joint SSFs (*knee^Flex-Exten^*, *hip^Flex-Exten^*, *knee^Abd-Add^*, and *knee^Int-Ext^*) at MS and TO. For SSV, we observed group differences only at TO (*ankle^Dorsi-Plantar^* and *hip^Abd-Add^* angles). The correlation analysis for elites revealed that, excluding *α^Time^*, both *α^Length^* and *α^Width^* were negatively correlated with SSV. Whereas for novices, *α^Time^* and *α^Width^* were positively correlated with SSV, while no significant correlation existed with *α^Length^* and SSV.

In this study, runners were asked to select the running speed at which they feel comfortable both kinematically and around the range of their submaximal intensity. Self-selection, rather than imposing a specific speed, has been reported to allow runners to maximize performance while using the least amount of energy [44]. Elite athletes ran at a considerably higher PRS than novice runners. Despite the differences in speed between novice and elite runners, no significant differences were found in RER for increasing time intervals. The RER values ranged between 0.75 and 0.85, indicating that both groups maintained submaximal level of their anaerobic threshold percentage throughout the run [24,32,33,45]. This ensures that the running biomechanics are comparable between groups and time intervals throughout the run.

Due to the redundancy in the human motor control system, even elites, whose motor abilities have been finely tuned via training, can exhibit intrinsic variability when a task is performed over multiple trials. During running, both the central tendency and variability of the end-effectors’ interactions with the ground, i.e., spatiotemporal parameters, is maintained at an adequate level by effectively modulating the joint kinematics [23]. In our study, we calculated the SSFs and SSV of step time, length, and width. We found no differences in *step-time^SSF^* or *step-length^SSF^*. In line with our study, Mo and Chow [21] found no group differences in mean stride interval between trained and untrained runners during prolonged running at their respective anaerobic thresholds. Still, they reported differences in time intervals across groups and considered them to be the result of fatigue. We found that both novice and elite runners maintain similar step-length throughout the run and also there were no significant differences between these groups. Previous studies have shown that regardless of the skill level, both elite and novice runners can select a stride length to maintain submaximal intensity level during running [24,45].

Although variability can also be perceived as noise, goal-directed variability demonstrates the motor system’s adaptability to internal and external stimuli [16,17,18]. In our study, the variability of step-length and step-width were significantly different between groups and were more variable in novices, whereas elite runners exhibited a trend of maintaining both. The timing of the occurrence of variability can also be important depending on which was affected earliest. In our study, group differences for variability in *step-width ^SSV^* at the 5~10 min period was followed by group differences in variability for *step-length* *^SSV^* at 10~15 min period which continued until the completion of the run, i.e., P4. In addition, we also observed differences in *step-width^SSF^* within groups as the time interval increased, where novices preferred to adopt relatively broader step-width despite running at a slower speed compared to elite runners. In running, step-width has been reported to be the primary mechanism for maintaining lateral balance, which accounts for ~2% of the energetic cost [46]. As a result, an increase in *step-width^SSV^* in a novice may indicate an increase in active control required to maintain lateral balance and may be a compensatory strategy for instability [46]. The modulation of step width also affects the running biomechanics. According to Brindle, et al. [47], increasing the width from narrow to broad has a significant effect on lower extremity biomechanics, decreasing the peak hip adduction angle, peak knee abduction moment, knee abduction impulse, and peak rearfoot eversion angle. Hence, the angular configuration is modified based on the interaction of end-effectors with the ground that generates the spatiotemporal parameters.

The running complexity evaluated using the alpha exponents of the spatiotemporal parameters did not differ between groups. The alpha exponents of all the stride parameters were larger than 0.5, suggesting the existence of long-range correlations [25]. Furthermore, our results are also consistent with Mo and Chow [21]; they examined the complexity of stride parameters for elite and novice runners during a prolonged run at their anaerobic thresholds. Although we did not observe differences in the group or time interval, the alpha exponent for the elites was smaller than that of novices until the 10 min time interval. Consistent with their findings, we also found a crossover between the alpha exponent of elite and novice runners after the 10 min time interval, supporting the notion of the existence of training-by-fatigue interaction on running complexity. These results highlight the importance of interpreting complexity in the context of associated control systems and biomechanical and neuromotor redundancies [21,48,49].

During the initial contact with the ground, i.e., heel strike, the angular configuration of the lower limb joints dictates the muscular demand to counterbalance the ground reaction force [50,51]. We found that elite runners had significantly higher hip flexion and knee extension angles than novice runners, whereas novice runners had significantly higher plantar-flexion angles. A study conducted by Kuitunen, et al. [52] found that trained sprinters use a running technique that regulates ankle and knee stiffness, as well as the importance of the triceps surae muscle-tendon unit of the ankle during running. Despite the fact that we were unable to measure muscular activity or tendon length changes, the change in plantar flexion angles seen only for the novices is likely due to a weaker triceps surae muscle-tendon unit than that of the trained athletes. Further, larger joint movements of proximal lower limb joints employed by elite runners may redistribute the muscular demand towards larger muscles to run a long distance with higher efficiency. Apart from this, larger hip flexion is also linked to an increase in the internal hip extension moments, which ensures that the runners stabilize their trunk and maintain balance while propelling their center of mass over the stance phase [53,54].

Midstance is a period of weight acceptance and preparation for take-off, during which knee angle has been demonstrated to play a role in stress absorption and propulsion during running. We found that novices showed significant differences in knee angle with the increase in time-interval. Novices ran with a lower flexion angle during midstance, suggesting joint angles that limit the capacity to absorb shocks during running. Previous research found that a greater knee flexion at contact lowered impact force but increased shock transferred to the shank [55]. Past studies reported that knee joint stiffness plays a vital role in controlling overall leg stiffness during running, and plays a key role in modulating the power required for push-off [52,56]. According to the spring-mass model, the length of the leg spring upon impact should be shortened, and the elastic energy created at this moment should be utilized for propulsion to improve RE. The reduction in knee angle for the novice over time suggests that energy generated during ground contact may not be effectively retained and used for propulsion, thus diminishing RE.

*Ankle^SSF^*, *knee^SSF^*, and *hip^SSF^* all exhibited an interaction at midstance, but only the *hip angle^SSF^* revealed a change at heel strike, the moment at which braking occurs during running. As a result, it is reasonable to conclude that the difference in joint kinematics at heel strike indicates the braking tactics utilized by the runners, which influence the kinematics of the subsequent phases, i.e., midstance and toe-off. This is confirmed using SSV analysis; we found that novices had high variability in *hip^Abd-Add^* angle and *ankle^Dorsi-Plantar^* at toe-off. Lees and Bouracier [57] reported that novice runners had higher variability in braking and propulsive impulses than experienced runners. Further, Hamill, et al. [58] also reported an increase in variability, which might be linked to running injuries. Hence, novice runners have high variability in hip rotation angle and are vulnerable to these risks.

The correlation analysis of *α^Time^* revealed a significant positive relationship with hip joint SSV for elites at HS and TO in the sagittal plane. Whereas, for novices, a positive correlation was observed during the MS and TO. As toe-off is also the preparatory phase for the upcoming heel strike, *hip^Flex^*^-Exten^ has been reported to help with shock absorption [37,59]. Mo and Chow [59] reported experienced runners employ effective hip strategy to adapt more favorably to fatigue than novices. Thus, lowering hip variability could be the strategy employed by elite runners to decrease the complexity related to time, i.e., increase the flexibility of the locomotor system. Overall, the *α^Length^* was negatively correlated for elite runners; however, this was not the case for novice runners. Particularly, both the knee and the ankle variability were negatively correlated at HS while only knee was negatively correlated at the MS and the TO. Although both expert and novice runners have been reported to be capable of adjusting their stride length to the most economical [24], economical runners tend to reduce ankle stiffness and increase knee stiffness to reduce oxygen consumption [60]. The negative correlation between knee joint kinematics and *α^Length^* may indicate a strategy used by trained runners to modulate stiffness in order to sustain higher speeds and RE. Similarly, *α^Width^* also exhibited a negative correlation with the joint SSV of elite runners, whereas it was positively correlated with joint SSV of novice runners. In particular, the positive association of *α^Width^* for novice runners with the knee joint SSV during TO and HS may imply our inherent control mechanism employed to maintain balance, i.e., a compensatory strategy for instability [46].

Several limitations need to be clarified and addressed in our study. First, the athletes were instructed to run at their PRS, which differed from their actual competition pace. Consequently, caution must be taken when developing training programs based on the study. Second, we focused on studying the differences in running mechanics at one’s PRS and did not directly estimate the exercise intensity based on the VO_2_max test but rather utilized RER as an indicator for the submaximal level of the anaerobic threshold. If exercise intensity needs to be considered, VO_2_max testing should be conducted separately to observe a plausible relationship between exercise intensity and running mechanics. Third, neither the muscle activity nor the ground reaction force were measured, which may be used to determine the internal joint forces during the prolonged run. Future studies are necessary to link the change in joint stiffness with increasing time intervals and may provide crucial information for enhancing running performance as well as the link with running-related injury mechanisms.

## 5. Conclusions

In this study, we assessed the effect of prolonged running on joint kinematics and stride complexity between novice and elite runners during prolonged running trials. With increasing time-interval, we observed a significant increase in the step width and length variability for novice runners compared to elite runners. Though we did not observe differences in the alpha exponent of spatiotemporal parameters, increased step-width variability could be a compensatory mechanism for novice runners to maintain performance and mitigate the loss of stability. Elite runners, on the other hand, showed a training-induced effective modulation of lower-limb kinematics to improve their running performance. The correlation of complexity with joint kinematic variability showed a distinct pattern for each group, more pronounced in elite runners with a negative association of kinematic variability with the stride complexity. On the other hand, contrasting results were observed for novice runners. Hence, we expect our findings to help coaches, therapists, and researchers to understand changes in kinematics and variability that are required to be targeted to monitor training-induced enhancement in marathon performance.

## Figures and Tables

**Figure A1:**
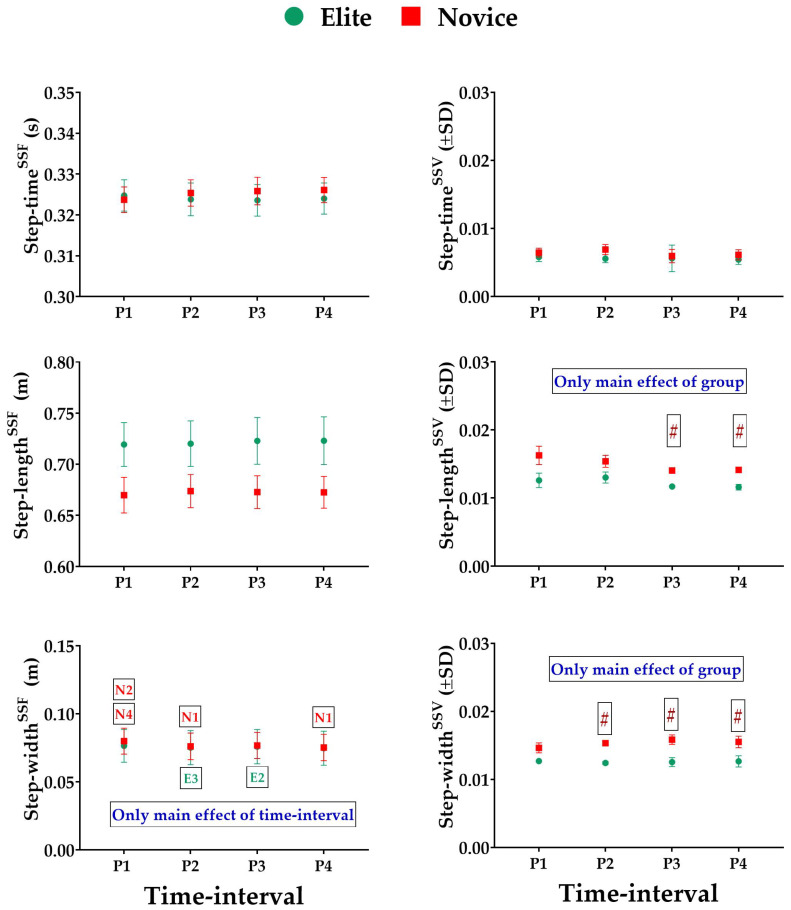
The results of SSF (**left**) and SSV (**right**) of spatiotemporal variables between elites (circle) and novices (square) in each phase of the prolonged run are shown. The computed variables are represented on the *Y*-axis. The time interval was computed by increasing the window size every five minutes, as seen on the *X*-axis. (P1: 0 to 5 min; P2: 0 to 10 min; P3: 0 to 15 min; and P4: 0 to 20 min). The graph depicts a statistically significant main effect of group, time interval, and interaction. The outcome of a pairwise comparison for the time-interval difference is represented by letters (N for novice and E for the elite) with the corresponding time-interval with a significant difference (e.g., N1 for the novices at time interval 0–5 min). Significant differences between groups for a specific time interval are marked with a “#”.

**Figure A2:**
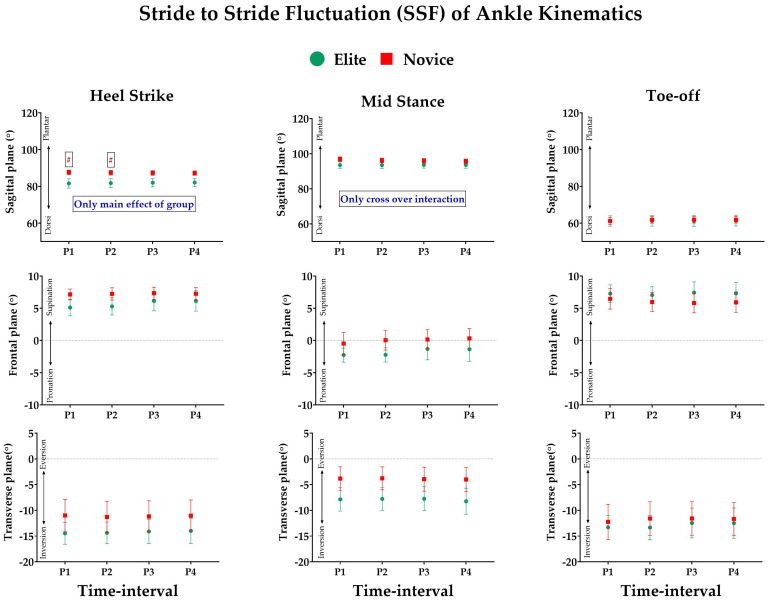
The results of the kinematics of ankle joint^SSF^ angle between elites (circle) and novices (square) in each phase of the prolonged run. The computed variable of SSF is represented on the *Y*-axis. The time-interval calculated by increasing the window size every five minutes is represented on the *X*-axis (P1: 0 to 5 min; P2: 0 to 10 min; P3: 0 to 15 min; and P4: 0 to 20 min). The figure depicts a significant main effect of group, time interval, and interaction. Significant differences in a group for a specific time interval are marked with a “#”. (Note: only results with significant interaction have been reported).

**Figure A3:**
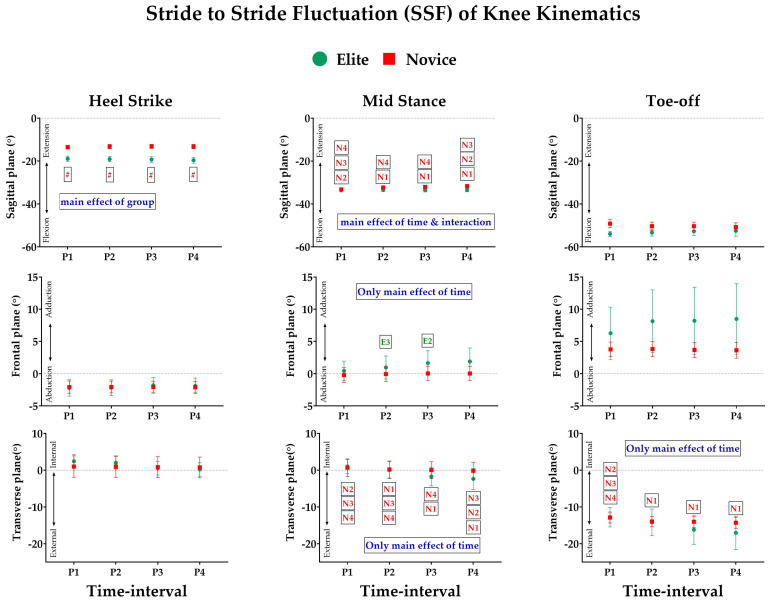
The results of the kinematics of knee joint^SSF^ angle between elites (circle) and novices (square) in each phase of the prolonged run. The computed variable of SSF is represented on the *Y*-axis. The time-interval calculated by increasing the window size every five minutes is represented on the *X*-axis (P1: 0 to 5 min; P2: 0 to 10 min; P3: 0 to 15 min; and P4: 0 to 20 min). The figure depicts a significant main effect of group, time interval, and interaction. The outcome of a pairwise comparison for the time-interval difference is represented by letters (N for novice and E for the elite) with the corresponding time-interval with a significant difference (e.g., N1 for the novices at time interval 0–5 min). Significant differences in a group for a specific time interval are marked with a “#”. (Note: only results with significant interaction have been reported).

**Figure A4:**
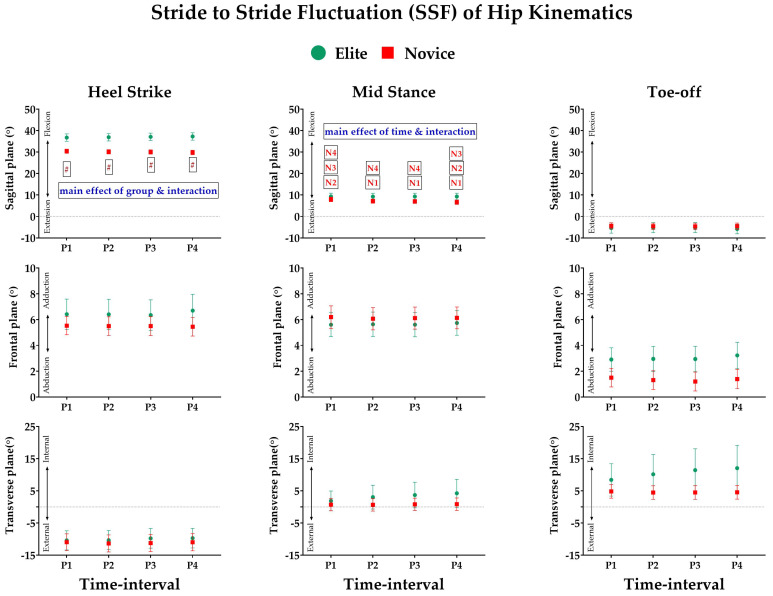
The results of the kinematics of hip joint^SSF^ angle between elites (circle) and novices (square) in each phase of the prolonged run. The computed variable of SSF is represented on the *Y*-axis. The time-interval calculated by increasing the window size every five minutes is represented on the *X*-axis (P1: 0 to 5 min; P2: 0 to 10 min; P3: 0 to 15 min; and P4: 0 to 20 min). The figure depicts a significant main effect of group, time interval, and interaction. The outcome of a pairwise comparison for the time-interval difference is represented by letters (N for novice with the corresponding time-interval with a significant difference (e.g., N1 for the novices at time interval 0–5 min). Significant differences in a group for a specific time interval are marked with a “#”. (Note: only results with significant interaction have been reported).

**Figure A5:**
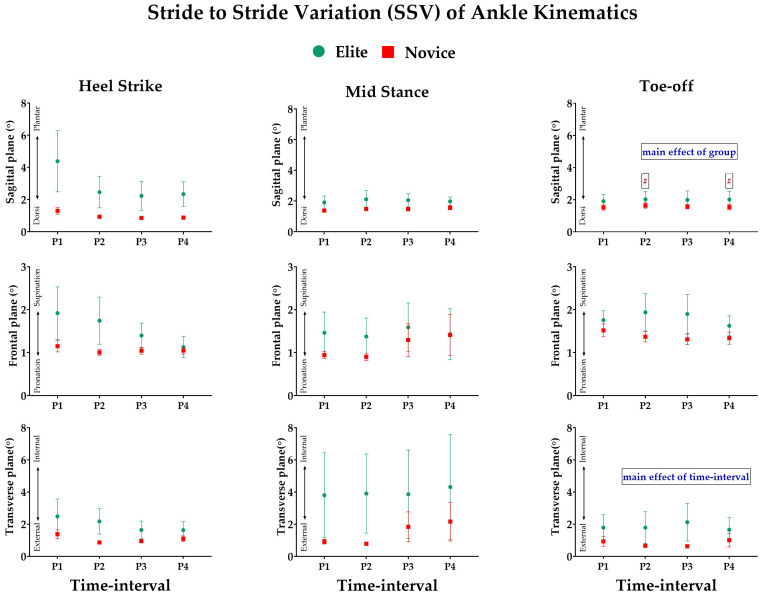
The results of the kinematics of ankle joint^SSV^ angle between elites (circle) and novices (square) in each phase of the prolonged run. The computed variable of SSV is represented on the *Y*-axis. The time-interval calculated by increasing the window size every five minutes is represented on the *X*-axis (P1: 0 to 5 min; P2: 0 to 10 min; P3: 0 to 15 min; and P4: 0 to 20 min). The figure depicts a significant main effect of group, time interval, and interaction. Significant differences in a group for a specific time interval are marked with a “#”. (Note: only results with significant interaction have been reported).

**Figure A6:**
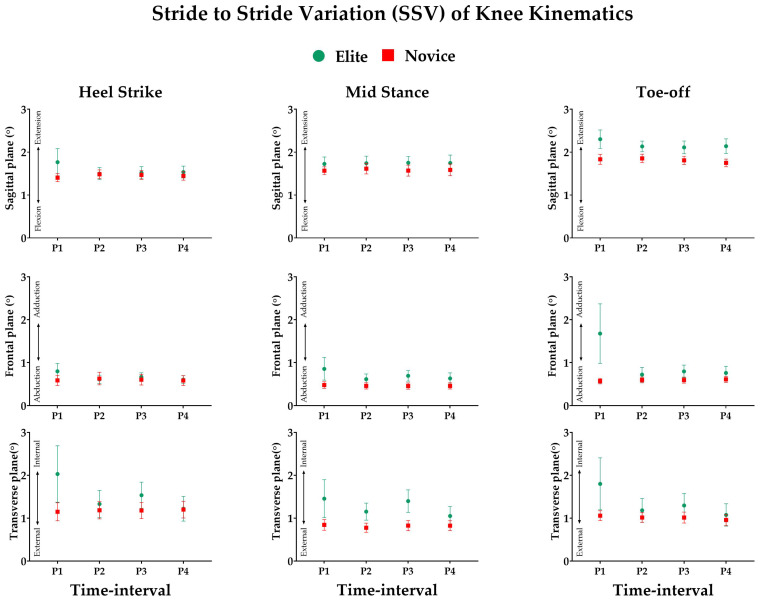
The results of the kinematics of knee joint^SSV^ angle between elites (circle) and novices (square) in each phase of the prolonged run. The computed variable of SSV is represented on the *Y*-axis. The time-interval calculated by increasing the window size every five minutes is represented on the *X*-axis (P1: 0 to 5 min; P2: 0 to 10 min; P3: 0 to 15 min; and P4: 0 to 20 min). The figure depicts a significant main effect of group, time interval, and interaction. (Note: only results with significant interaction have been reported).

**Figure A7:**
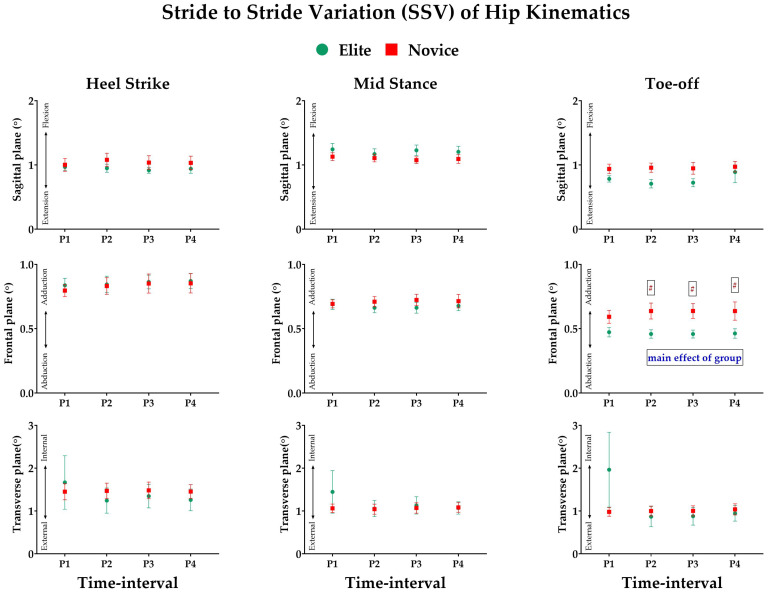
The result of the kinematics of hip joint^SSV^ angle between elites (circle) and novices (square) in each phase of the prolonged run. The computed variable of SSV is represented on the *Y*-axis. The time-interval calculated by increasing the window size every five minutes is represented on the *X*-axis (P1: 0 to 5 min; P2: 0 to 10 min; P3: 0 to 15 min; and P4: 0 to 20 min). The figure depicts a significant main effect of group, time interval, and interaction. Significant differences in a group for a specific time interval are marked with a “#”. (Note: only results with significant interaction have been reported).

## Data Availability

Data are available upon request from the corresponding author of the study.

## Appendix A

**Table A1:** Descriptive information of each participant.

S.N	CODE	Age (Years)	Mass (kg)	Height (m)	PRS (m/s)	Half Marathon Record (hh:mm)	Experience (Years)
1	S#E01	34	50	1.65	3.75	1:21	3
2	S#E02	23	56	1.71	3.89	1:10	11
3	S#E03	33	68	1.72	3.47	1:22	5
4	S#E04	28	67	1.72	4.03	1:19	4
5	S#E05	26	61	1.73	3.94	1:10	10
6	S#E06	23	65	1.76	3.86	1:07	13
7	S#E07	27	78	1.77	3.64	1:22	3
8	S#E08	26	66	1.82	3.56	1:10	14
9	S#E09	30	83	1.87	3.89	1:14	10
10	S#E10	30	68	1.88	3.83	1:06	13
Elite	Mean	28	66	1.76	3.79	1:14	8.60
	[M71] SD	4	10	0.07	0.18	0:06	4.40
1	S#N01	20	77	1.83	2.86	N/A	N/A
2	S#N02	20	69	1.84	3.06		
3	S#N03	22	75	1.74	3.06		
4	S#N04	23	62	1.71	2.61		
5	S#N05	24	81	1.9	2.92		
6	S#N06	24	76	1.8	2.92		
7	S#N07	25	73	1.73	2.25		
8	S#N08	25	83	1.74	2.53		
9	S#N09	25	79	1.87	3.06		
10	S#N10	26	68	1.77	3.06		
11	S#N11	31	89	1.85	2.78		
Novice	Mean	24	76	1.80	2.83	N/A	N/A
	SD	3	8	0.06	0.26		

## Appendix B

### Appendix B.1. Estimation Procedure of Preferred Running Speed (PRS)

First, for the references, the athletes were asked to report their PRS as the speed that best defines their mean speed during running competitions. Likewise, novice runners were asked to report their PRS as the speed that best defines their treadmill speed while performing aerobic exercise at the gym. Although, we were able to find a study that reported a non-significant effect of the use of indirect calorimetry system on joint kinematics [19]. In order to avoid differences in perceived intensity or kinematics during PRS estimation and actual test, components of indirect calorimetry system were attached to the subject which included a mouthpiece, nose clip, headgear, wireless sensor, and heart rate (HR) sensor.

The PRS estimation procedure started with the subjects running at a self-selected speed on the treadmill for 2 min. The speed was then gradually increased by the experimenter at 0.1 km/h. until the subject reported the running speed that best defined their PRS. The speed was increased by 2 km/h. and slowly decreased by 0.1 km/hr. until the subject reported that the running speed that best defined their PRS. During the estimation of PRS, the treadmill screen was covered to avoid bias in the reporting of the speed [25]. The process was repeated three times, and the mean value was taken as the PRS. The subjects were asked to report their PRS only if they felt that they had a comfortable stride length and stride rate for a given speed. To ensure the reliability of the measurement, their HR and RER were continuously monitored by the experimenter to be within 70% to 80% of the maximum HR and RER in the range 0.70~1.0. After determining the PRS, subjects performed familiarization at their respective speed for at least 5 min and rested for at least 10 min.

### Appendix B.2. Calculation of Submaximal Level of Anaerobic Threshold Percentage Using Respiratory Exchange Ratio (RER)

The RER ratio was computed as an indicator to ensure that the submaximal level of the anaerobic threshold (0.7~1.0) was maintained [32,33]. Exceeding the anaerobic threshold can cause fatigue due to the accumulation of lactate and affect the running mechanics. During the experiment, the level of change in RER was visually monitored. For further analysis of the raw data from the indirect calorimetry device, a zero-lag 4th order Butterworth low pass filter at 0.04 Hz was applied with custom Matlab Script to attenuate the variability in the data acquired using a breath-by-breath protocol [61]. The oxygen consumption ($\dot{V}O_{2}$) and carbon dioxide production ($\dot{V}CO_{2}$) were calculated based on the timestamp of the raw data. The net ($\dot{V}O_{2}$) and net ($\dot{V}CO_{2}$) during running were calculated by subtracting the gross ($\dot{V}O_{2}$) and ($\dot{V}CO_{2}$) while sitting in a steady state. Thereafter, the following equation was used to calculated RER.

(A1) $${\mathrm{RER}}_{time-intervals}=\left(\frac{\dot{V}CO_{2}gross}{\dot{V}O_{2}\;gross}\right)$$

### Appendix B.3. Details on Marker Attachment Location

- Joint markers: left/right anterior superior iliac spine, left/right posterior superior iliac spine, left/right great trochanter, left/right femur lateral epicondyle, left/right femur medial epicondyle, left/right fibula apex of the lateral malleolus, left/right tibia apex of the medial malleolus, left/right posterior surface of the calcaneus, left/right head of the 5th metatarsus, left/right proximal medial phalanx, left/right head of the 1st metatarsus.
- Tracking clusters markers: three tracking markers for each segment of the thighs (left/right) and shanks (left/right).
